# Computational search for UV radiation resistance strategies in *Deinococcus swuensis* isolated from Paramo ecosystems

**DOI:** 10.1371/journal.pone.0221540

**Published:** 2019-12-02

**Authors:** Jorge Díaz-Riaño, Leonardo Posada, Iván Camilo Acosta, Carlos Ruíz-Pérez, Catalina García-Castillo, Alejandro Reyes, María Mercedes Zambrano

**Affiliations:** 1 Corporación Corpogen Research Center, Bogotá D.C, Colombia; 2 Research group in Computational Biology and Microbial Ecology, Department of Biological Sciences, Universidad de Los Andes, Bogotá D.C, Colombia; 3 Max Planck Tandem Group in Computational Biology, Universidad de Los Andes, Bogotá D.C, Colombia; 4 Center of Genome Sciences and Systems Biology, Washington University School of Medicine, Saint Louis, MO, United States of America; Bhabha Atomic Research Centre, INDIA

## Abstract

Ultraviolet radiation (UVR) is widely known as deleterious for many organisms since it can cause damage to biomolecules either directly or indirectly via the formation of reactive oxygen species. The goal of this study was to analyze the capacity of high-mountain *Espeletia hartwegiana* plant phyllosphere microorganisms to survive UVR and to identify genes related to resistance strategies. A strain of *Deinococcus swuensis* showed a high survival rate of up to 60% after UVR treatment at 800*J*/*m*^2^ and was used for differential expression analysis using RNA-seq after exposing cells to 400*J*/*m*^2^ of UVR (with >95% survival rate). Differentially expressed genes were identified using the R-Bioconductor package NOISeq and compared with other reported resistance strategies reported for this genus. Genes identified as being overexpressed included transcriptional regulators and genes involved in protection against damage by UVR. Non-coding (nc)RNAs were also differentially expressed, some of which have not been previously implicated. This study characterized the immediate radiation response of *D. swuensis* and indicates the involvement of ncRNAs in the adaptation to extreme environmental conditions.

## Introduction

Diverse natural and artificial environments exposed to extreme temperature, pressure and/or radiation conditions are attractive sources of microorganisms with exceptional phenotypic and genotypic properties. The high-mountain Paramo biome, similar to the tundra biome of high latitudes, consists of high-elevation areas subject to harsh environmental conditions. The Paramo biome has a high solar incidence that can induce damage by ultraviolet radiation (UVR) that represents a survival challenge for organisms [1]. Ionizing radiation and UVR affect organisms by damaging cellular components such as nucleic acids, proteins, and lipids [2]. The deleterious effect on cells is caused by direct damage to DNA, such as chromosomal lesions that introduce both double-strand breaks (DSBs) and single-strand breaks (SSBs), and damage due to pyrimidine dimerization and photoproducts that inhibit DNA replication and transcription [3]. Most of the damage, however, is caused indirectly by the production of reactive oxygen species (ROS), such as the chemically reactive superoxide and hydroxyl radicals that in turn affect various cellular constituents, including proteins [2].

The harmful effects of UVR on cellular components depend on the wavelength: UVA can travel farther into tissues and contributes to ROS (damage to lipids, proteins, and DNA) whereas UVB produces direct breaks in the DNA structure (pyrimidine dimers) [4]. Even though UVC radiation is not present on the Earth’s surface, its bactericidal potential is used for studying UV sensitivity in bacteria with a high tolerance to UVB or UVA radiation [5]. Organisms resistant to radiation have been identified in all three domains of life. The mechanisms proposed to be involved in resistance to UVR vary and include strategies for DNA repair, protection against ROS using either enzymes or non-enzymatic antioxidative defenses, such as intracellular manganese and pigment production, protein folding and degradation systems [6]. Bacteria, with their diverse metabolic capacity, have an uncanny ability to survive under extreme conditions and colonize habitats that are inhospitable to other groups of organisms [7]. Different levels of resistance to UVR have been reported in diverse bacterial species, highlighting a wide variation in the response and a need for understanding the physiological, biochemical and mechanical responses that confer UV tolerance in bacteria [8]. Perhaps the most representative members of the extremely radiation-resistant bacteria belong to the family Deinococcaceae, which can survive exposure to ionizing radiation over 12,000 Gy (J/Kg), UVR over 1000*J*/*m*^2^ and can grow under harsh chronic irradiation of 50 Gy per hour [9]. *Deinococcus swuensis*, whose genome was recently published from a strain isolated from soil in South Korea, is also reported to have high resistance to UVR [10, 11].

Transcriptomic studies of *D. radiodurans* under radiation stress have shown induction of genes involved in DNA repair, cell recovery and antioxidative defenses [12]. An RNA-Seq analysis of *D. gobiensis* also showed induction of genes for DNA repair and regulation in response to UVR [13]. These studies, together with more recent work [14], also indicate differential expression of a subset of small and noncoding RNAs (sRNAs/ncRNAs), molecules that do not encode functional proteins but can play important roles in regulation of transcription and translation [15].

The differential expression of ncRNAs, upon UVR treatment suggests that these molecules could be important in triggering protective mechanisms, even though their precise role during the stress response to high doses of radiation still remains to be determined. A new hypothesis suggests that sRNAs could contribute to cellular post-exposure recovery because they would remain largely undamaged due to their small size [14]. Experimental evidence places these sRNAs into different metabolic pathways, such as response to changes in temperature, pH and other lethal stressors [16]. Recently reported sRNAs identified to be involved in radiation resistance are Y-RNAs, molecules that adopt specific secondary structures and bind to proteins known as Ro that are conserved in several organisms [17]. In *D. radiodurans* Y-RNAs were found to bind the Ro orthologue Rsr to form a ribonucleoprotein (Ro-RNP) complex that functions as an effective machinery for bacterial RNA degradation [18]. *D. radiodurans* was found to upregulate and accumulate Ro-RNPs in response to UVR and cells lacking the Ro protein had decreased survival following UV exposure [19].

In this study we hypothesized that microorganisms capable of resisting UVR should be present in locations exposed to high solar incidence, such as the Andean mountain high-altitude Paramo biome. Previous results indicated that the phyllosphere microbiota associated with *Espeletia sp*., a plant endemic to the Paramo, contained diverse microbial communities and genes involved in resistance to UV and other stress conditions [1], and could thus provide insight into microbial resistance strategies. The main goal of this work was to isolate UV resistant microorganisms from this plant phyllosphere and study their resistance mechanisms through gene expression analysis. One bacterial strain identified as *D. swuensis* showed high resistance to UV exposure in laboratory settings and differential regulation of genes and sRNAs that provide clues to the immediate response of *D. swuensis* to radiation and extreme environmental conditions, such as those found in high Andean ecosystems.

## Materials and methods

### Isolation of bacterial strains, culture conditions and characterization

Microorganisms were isolated from *Espeletia hartwegiana* leaves were collected at the National Natural Park Los Nevados in Colombia (04°52’27” N; 75°15’51.4” W), as previously described [1]. The sampling was done under MADS contract no. 76-2013 for access to genetic resources. Microbes were first dislodged from leaf surfaces, as reported [20], and then plating serial dilutions on R2A Agar (BD Difco, Franklin Lakes, NJ) and Tryptone soy agar (TSA, Oxoid), supplemented with 50 mg/ml Nystatin (Sigma-Aldrich, St. Louis, MO) to avoid fungal growth, when necessary. Plates were incubated at 25°C for 15 days and checked daily for growth. Colonies with distinct morphologies were re-streaked in the same growth media until pure colonies were obtained. Strains were characterized microscopically using Gram staining and taxonomic identification was done by analysis of the 16S rRNA gene or the ITS region for fungi. DNA was obtained by resuspending colonies in 1ml Tris 10mM (pH 8.0), adding 25*μ*l proteinase K (10mg/ml) and incubating at 55°C for 25 min. DNA was purified from 500*μ*l of this cell suspension using the MO BIO Microbial Ultraclean DNA Purification Kit (Qiagen, Germany). PCR amplifications were done using primers 27F (5’ AGAGTTTGATCMTGGCTCAG 3’) and 1492R (5’ TACGGYTACCTTGTTACGACTT 3’) for bacteria, in a 50*μ*l reaction volume containing 1*μ*l DNA template, 0.2*μ*M of each primer, 0.2 mM dNTPs, 2.5 mM MgCl_2_, 1X Buffer and 1.25 U of Taq DNA polymerase (CorpoGen, Colombia) and the following amplification conditions: 4 min at 94°C, 35 cycles of 30 s at 94°C, 45 s at 55°C, 1 min a 72°C, and a final extension of 10 min at 72°C. Primers ITS5 (5’ GGAAGTAAAAGTCGTAACAAGG 3’) and ITS4 (5’ TCCTCCGCTTATTGATATGC 3’) were used to amplify fungi as described above but using 0.3 *μ*M primers and PCR reactions of 2 min at 94°C, followed by 35 cycles of 60 s at 94°C, 60 s at 55°C, 1 min a 72°C, and a final extension of 5 min at 72°C. Sequencing was performed in an Applied Biosystems 3500 Genetic Analyzer. Forward and reverse reads were assembled and analyzed using Geneious 8.2, removing low quality nuclotides, and queried against the NCBI nucleotide database using BLAST.

### Screen for UV resistance and *D. swuensis* survival curve

Strains were grown overnight in 3ml Tryptone soy broth (TSB, Oxoid), washed 3 times with PBS, and 20*μ*l of nine 1:10 serial dilutions were spotted, in triplicate, on TSA medium, allowed to dry, and exposed to UVC in a UV hood to obtain a fluence rate from 50 to 800*J*/*m*^2^, as previously described [21] and determined using a radiometer with an LP 471 UVC probe (Delta Ohm, HD2302.0). Survival was determined by plating irradiated cultures on TSA medium to determine CFU/ml. Survival of *D. swuensis* was measured at various points along the growth curve using three replicate cultures that were first grown overnight and then diluted 1:100 in 100 ml fresh TSB medium, and incubated at 30°C, with continuous agitation at 150rpm. Samples were taken at 15, 24, 40, 48, and 72 hours and exposed to 800, 1600 and 2400*J*/*m*^2^ to determine survival (CFU/ml), as mentioned above.

### RNA extraction and sequencing

Triplicate 48-hour *D. swuensis* cultures were grown first for 48h in 3ml TSB, then diluted 1:100 into 100 ml fresh medium and re-grown for 24h. Ten ml of each 24h culture (OD ≅ 1; approximately 1.6*x*10^8^ *CFU*/*ml*) were transferred to a sterile Petri dish and submitted to 400*J*/*m*^2^ irradiation. After exposure, bacterial cells were immediately placed on ice, and centrifuged at 4600 x g for 15 minutes (4°C). Control aliquots from the same culture were not submitted to irradiation. After centrifugation, pellets were re-suspended in 1ml TriZol (Promega), lysed with Matrix B lysing beads (MP Biomedicals) in a FastPrep (MP Biomedicals) using 6.5 m/s for 40 seconds, and then centrifuged at 15,000 x g for 1 minute at 4°C. RNA in supernatants was recovered with the DirectZol RNA extraction kit (Zymo Research). Only RNA with a RIN >8 was used for sequencing at Macrogen (Seoul, Korea) on an illumina Hi-seq 2000, with 100 nucleotide paired-end reads.

### Preprocessing and mapping sequencing data

Quality control was made with FastQC (v.0.11.2) (http://www.bioinformatics.bbsrc.ac.uk/projects/fastqc/), Illumina adapters were trimmed with Trimmomatic (v.0.36) [22], rRNA depletion was performed with SortmeRNA (v.2.1) [23] using the Silva16S, 23S and 5S rRNA gene databases (release 128 downloaded on January 2017 from https://www.arb-silva.de/no_cache/download/archive/release_128/) [24]. Sequences were mapped against the *D. swuensis* NCBI reference genome DY59 (accession number: GCF_000800395.1) [11] with Subread (V1.5.0-p3) [25], parameters included an insert size of 250 bp, a maximum of 3 mismatches and 5 indels. Features present in the reference annotation were extracted from the gff file and relative abundances were calculated with featureCounts, a tool included in the R package subread (v.1.5.0-p3) [26], and an in-house script. Remaining (unmapped) sequences were randomly subsampled (10%) and searched against the nt (nucleotide collection) database with Blastn and processed with MEGAN (v.6.9.4) at a threshold of 1*X*10^−5^ [27].

### Differential expression analysis

Annotated features from the reference genome such as coding DNA sequences (CDS’s), ncRNAs, pseudogenes, rRNAs and tRNAs were selected for analysis. The R-Bioconductor package NoiseqBio (2.18.0) [28] was used to measure differential gene expression between irradiated and non-irradiated control conditions. The workflow included a variance diagnostic (Cochran C test), analysis of sequencing depth, search for biases due to i) feature length and RNA amount and ii) detection of features with low counts, and the nonparametric analysis of differentially expressed features (based on Bayesian statistics). Counts were normalized to reads per kilobase of feature length per million mapped reads (RPKM) and by trimmed mean of M-values (TMM). Filtering of features with low counts was applied in order to remove those features that had an average expression of less than 5 CPM (counts per million) per condition and a variation coefficient higher than 100 in all conditions, which introduces noise and can lead to unreliable results for differential expression analysis [29]. According with developers suggestion, genes with a cut-off probability of expression above 0.8 and a log_2_ fold-change greater than or equal to 1.0 were considered as differentially expressed genes [28]. Sequences coding for annotated hypothetical proteins were queried against the NCBI nr database using BLASTp and used for domain search with HMMER (V.3.1) [30] against the PFAM database [31].

### ncRNA computational analysis

Intergenic regions of the reference genome showing a significant number of mapped transcriptomic reads (minimum 6X coverage) were retrieved as potentially containing ncRNAs. Filters for the regions selected were based on the number of hits (read counts) and the region length (>50pb). Candidate regions were compared against the Rfam and NCBI nucleotide-nr databases [32, 33] using covariance models implemented in Infernal (V.1.1) [34]. All ncRNA candidates were processed for differential expression analysis using the workflow described above.

### Quantitative real time PCR (qRT PCR) validation

Primers were designed using the IDT primerQuest tool (https://www.idtdna.com/Primerquest/Home/Index) to have a TM = ∼60°C, a final amplified product size of ∼200pb and GC content ∼50% S1 Table. RNA samples were quantified using a Qubit fluorometric system (Invitrogen) and used at the same concentrations for cDNA synthesis using Super script III reverse transcriptase (Invitrogen). qPCRs were run in a LightCycler ^®^ 96 System (Roche) using the FastStart Essential DNA Green Master kit (Roche) and the following conditions: 1 cycle of 600 s at 95°C, then 45 cycles of 10 s at 95°C, 10s at the annealing temperature and a final extension at 72°C for 10s; a melting curve after the amplification confirmed a single peak and indicated a specific qPCR product. Relative expression was obtained by normalizing with the single copy gene QR90_RS09970 that codes for a succinate dehydrogenase, that showed similar expression levels among the different samples and conditions in the RNA-seq analysis, and the equation proposed by [35]. Primer efficiencies were determined using 1:10 serial dilutions of genomic *D. swuensis* DNA and the same PCR program described above.

## Results

### Strain isolation and radiation resistance

**Fig 1:**
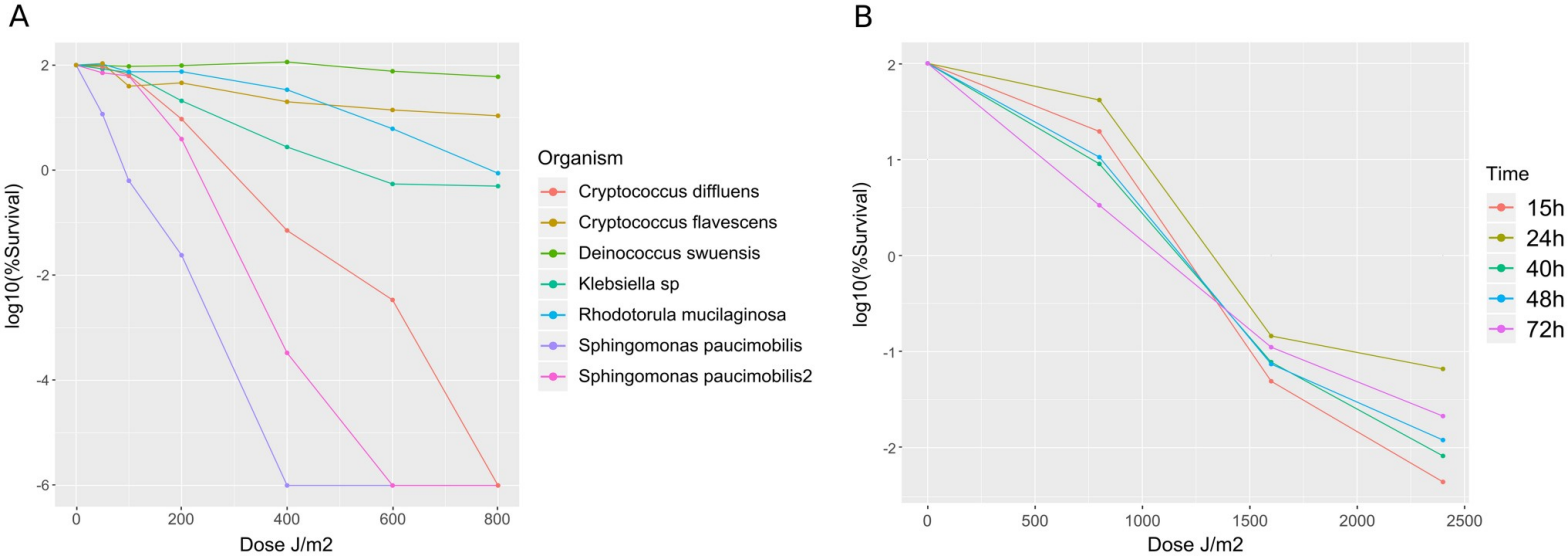
Survival to UV-C exposure of *Espeletia* phyllosphere-associated microorganisms. **(A)** Bacteria and yeast isolated from the plant phyllosphere were exposed to different UVR doses. Survival was measured as the percentage of CFUs obtained when compared to unexposed cells of the same strain. **(B)** Survival (mean±SD, n = 3) of *D. Swuensis* harvested at different time points along the growth curve (15-72h) and exposed to different doses of UV-C.

Microorganisms were isolated from the phyllosphere of *Espeletia* plants located in the National Park Los Nevados in Colombia [1]. Isolates with distinct colony morphologies were obtained by plating dilutions of the material dislodged from leaf surfaces on various media. Taxonomic identification using both 16S rRNA gene and ITS sequence analyses showed that this collection of isolates consisted of 10 fungi, 11 Gram-positive and 29 Gram-negative bacteria. To determine if any of these strains were resistant to UV radiation, as predicted for organisms living at these high-altitude ecosystems [1], all isolates were subjected to irradiation with UVC. A screen using varying levels of exposure, up to 800*J*/*m*^2^, showed that very few strains were capable of surviving these conditions. The most resistant strain was a bacterium identified as *D. swuensis* (strain CG1225), followed by the fungi *Cryptococcus flavescens* and *Rhodotorula mucilaginosa* Fig 1A. Other isolates showed reduced levels of resistance. Given that *D. swuensis* CG1225 showed the highest resistance to UVC exposure, with >60% survival at the highest dose tested (800*J*/*m*^2^), this strain was selected to further study its response to irradiation using RNAseq analysis.

In order to determine the best conditions for RNA extraction, a survival curve was first performed by harvesting *D. swuensis* cells at five different times along the growth curve and exposing these cells to varying doses of UVC, including doses above 800*J*/*m*^2^ used previously Fig 1B. Radiation resistance was similar for all time points examined along the growth curve (15 h to 72 h cultures), even up to the maximum exposure tested (2400*J*/*m*^2^). However, degradation of the extracted RNA was observed at increased doses of UV exposure. Thus we selected the treatment of 400*J*/*m*^2^ with cells grown for 24 h for subsequent RNA extractions to ensure sufficient recovery of high-quality RNA.

### Pre-processing and mapping sequencing data

Total RNA was obtained for three independent replicates of unexposed controls (C1, C2, and C3) and irradiated cultures (IR1, IR2, and IR3). RNA-seq was carried out using 100-nucleotide paired-end sequencing on an Illumina HiSeq. On average, 16.4 million reads were obtained per sample Table 1. After quality processing and adapter removal, rRNA filtering was performed using the SILVA database, which on average removed 90% of the reads, with the exception of samples C2 and IR2 for which 95% and 43% of the reads were retained, respectively, likely due to variation in the efficiency of experimental rRNA depletion [36]. Given the high number of reads retained after filtering for samples C2 and IR2 Table 1, a Cochran C test was performed to estimate significant differences in variance for any sample with respect to the entire group variance. The sample variance for C2 (0.9226) was significantly higher than the variance for the other samples (p-value of 2.2e-16) S2 Table, potentially leading to biases. In consequence, the C2 sample was removed from subsequent analyses. For the IR2 sample, in which 43% of reads were retained, the calculated variance (0.6849) was not significantly different from the other samples.

**Table 1:** Reads counts (in millions) per sample through the preprocessing and mapping pipeline.

Category	Features^1^	C1^2^	C2	C3	IR1	IR2	IR3
**Raw reads**		15.360	16.830	16.060	16.200	16.530	17.330
**After adapter removal**		15.360	16.820	16.050	16.200	16.520	17.320
**After rRNA removal**		1.820	16.090	2.290	1.300	6990	1.140
**Unmapped**		0.160	1.460	0.220	0.110	0.660	0.110
**Mapped**	3223	1.660	14.630	2.070	1.190	6.330	1.030
*CDS*^3^	3168	1.044	10.804	1.376	0.697	5.251	0.620
*ncRNA*^3^	1	0.079	0.539	0.090	0.049	0.299	0.034
*rRNA*^3^	7	0.036	0.005	0.045	0.059	0.034	0.073
*tRNA*^3^	47	0.004	0.123	0.005	0.005	0.010	0.003
*Unassigned*^3^		0.496	3.159	0.553	0.380	0.735	0.300

^1^ Number of features identified in the annotated genome.

^2^ Values in Millions of reads.

^3^ Features belonging to mapped category.

Sequences were mapped against the reference *D. swuensis* genome DY59. The percentage of mapped reads ranged between 90.55% and 91.38%, with a maximum of 3 allowed mismatches Table 1. This range is expected when mapping against a different strain of the same species, due to intraspecific variability [13, 14, 37]. Features annotated as CDS, ncRNA, rRNA, and tRNAs were extracted from the dataset for differential expression analysis. The majority of the mapped reads corresponded to CDS (67.48 ± 7.27%; mean ± SD) distributed among 3168 genes. A total of 4.15 ± 0.46% of the remaining reads mapped to a single ncRNA, making this the highest-scoring single feature. rRNA (7 features; 2.82 ± 2.12%) and tRNAs (47 features; 0.37 ± 0.15%) showed lower counts. Approximately 25.15 ± 5.69% of mapped reads could not be assigned to any annotated feature Table 1.

As can be seen in Table 1, on average 10% of the reads failed to map against the reference genome. To identify the putative origin of these sequences, 10% of the unmapped reads (182,615 for controls and 86,952 for irradiated samples) were queried against the NCBI non-redundant (nr) nucleotide database using BLASTn. A total of 61,151 (33.49%) and 31,607 (36.35%) reads for controls and irradiated samples, respectively, were identified as having significant hits to the database (with an e-value threshold of 1e-5). Taxonomic assignment of the BLASTn results examined using MEGAN showed that for both controls and irradiated samples, ∼31.15 ± 0.19% of the reads corresponded to *Deinococcus*-related bacteria, another ∼65.08 ± 2.025% had no hit to the database, and the remaining 3.98 ± 0.18% were assigned to other bacterial groups S1 Fig. The fact that ∼30% out of the 10% unmapped reads were assigned to other *Deinococcus* species suggests intraspecific strain variation, in concordance, the alignment of these reads against reference genome DY59 (through Blast) recovered matches associated to described protein and RNA metabolism with identity values over 85.

### Differential expression analysis

**Fig 2:**
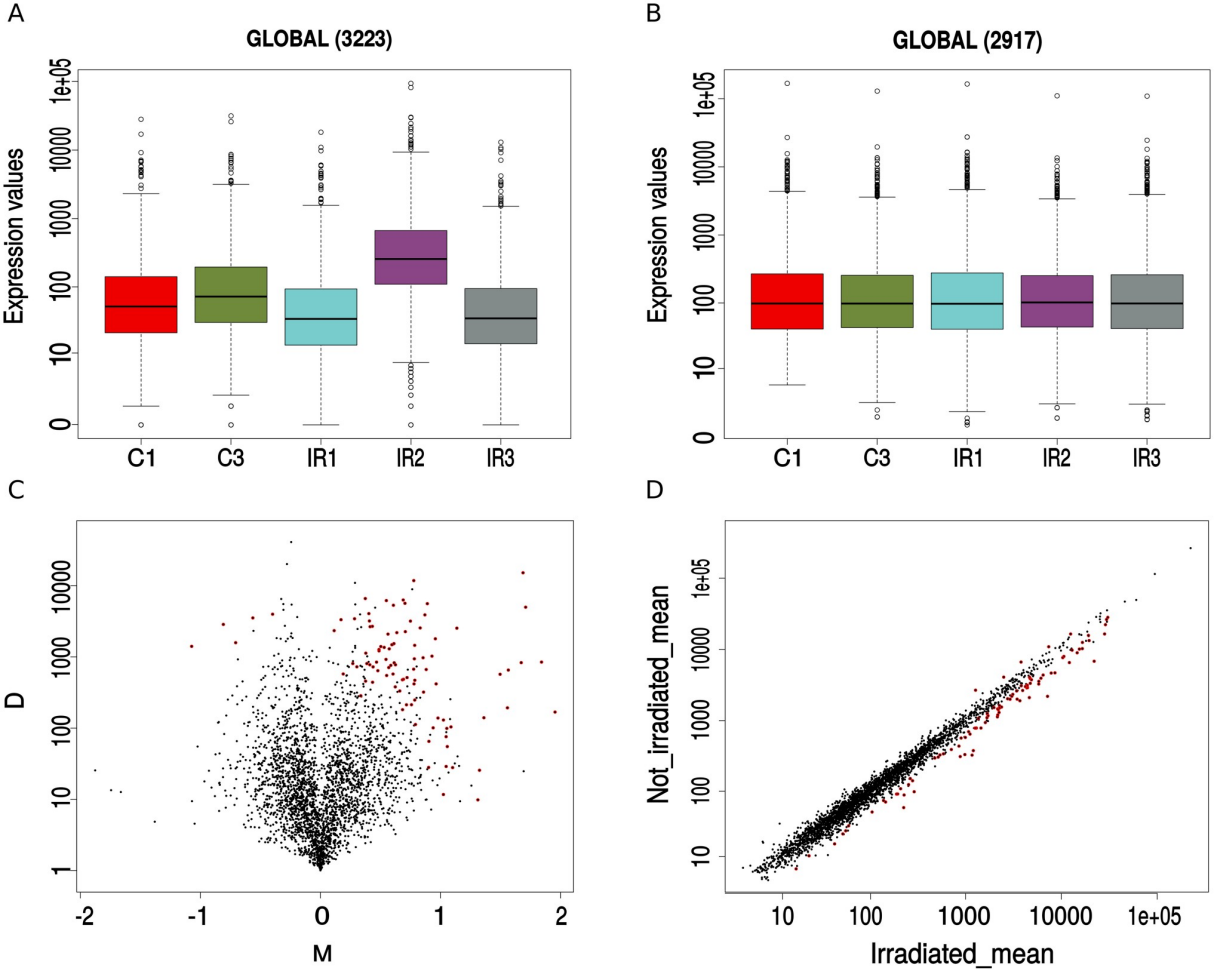
Differential expression analysis between irradiated and control samples. Boxplots showing expression values (in counts per million) for control (C1, C3) and irradiated (IR1-IR3) samples before **(A)** and after **(B)** the filtering of low counts (CPM <5) and the normalization process done by RPKM (reads per kilobase of feature length per million mapped reads) and TMM (trimmed mean of M-Values). **(C)** Volcano plot of log-fold change (M) vs. the absolute value of the difference in expression between conditions; genes with a bayesian posterior probability of differential expression >0.8 are shown in red, values of M >0 represent upregulated genes. **(D)** Correlation plot between irradiated (x-axis) and control (y-axis) mean expression. Genes deviating from expected with a probability >0.8 are shown in red. Values below and above the diagonal represent differentially expressed genes for the irradiated condition.

To identify genes potentially involved in resistance to UV exposure, differential expression analyses were performed using all identified genomic features (CDS, ncRNA, rRNA, and tRNA) using control (C1 and C3) and irradiated samples (IR1, IR2 and IR3). Because an independence assumption is required to obtain accurate conclusions, it is essential to minimize external factors that could affect gene expression, regardless of the experimental condition being tested. The data were therefore first filtered by removing low count features (less than 5 CPM [counts per million]) and normalized by 1) sequencing depth and feature length variation (RPKM), and 2) taking into account sample total RNA content using the TMM method, (Trimmed Mean of M values is the average expression value after removing the most variant features of the data); this normalization takes into account sample-to-sample variation Fig 2A and 2B.

A total of 96 differentially expressed features with log_2_ fold-change values ranging between -1.07 and 1.95 and a posterior probability for differential expression (p) >0.8 (NOISeq uses a bayesian approach to calculate the differentially expressed genes) were obtained Fig 2C and 2D. The chromosomal location of features that were up or down regulated did not show a particular position bias. The 96 detected features corresponded to four rRNAs, 13 tRNAs and 79 CDS (23 were hypothetical proteins), but only 14 CDS presented log_2_ fold-change values >1, indicating an over expression of at least twice as much as the control condition. Of these, 10 had functional annotation and 4 were hypothetical-proteins Table 2. The over-expressed CDS included genes for a GntR-like bacterial transcription factor, a proline dehydrogenase (key gene for homeostasis and ROS control in cells), RNA helicase (involved in ribosome biogenesis, initiation of translation), CrcB (protein for transmembrane transport of fluoride), an alpha/beta Hydrolase, a GTP-binding protein, Hemolysin, an ABC transporter ATP-binding protein and a pyr operon involved in synthesis of pyrimidines.

**Table 2:** Differentially expressed genes.

GenID^1^	I_mean^2^	NI_mean^2^	Theta^3^	Prob^4^	Log_2_FC^5^	Function
QR90_RS11755	224.78	57.99	0.98	0.92	1.95	GntR
QR90_RS02510	984.31	332.59	1.24	0.9	1.57	Hypothetical protein
QR90_RS11750	289.78	98.42	0.92	0.85	1.56	Proline dehydrogenase
QR90_RS09640	226.93	88.31	1.11	0.92	1.36	RNA helicase
QR90_RS15645	40.86	16.31	0.87	0.8	1.33	CrcB protein
QR90_RS08275	14.76	5.95	0.65	0.81	1.31	Alpha/beta hydrolase
QR90_RS05520	4636.4	2108.59	1.58	0.98	1.14	30S ribosomal protein S8
QR90_RS09935	50.29	23.44	0.73	0.8	1.1	Hypothetical protein
QR90_RS15125	194.2	91.37	0.82	0.84	1.09	Hypothetical protein
QR90_RS06220	103.97	49.97	0.66	0.82	1.06	GTP-binding protein
QR90_RS15620	54.06	26.16	0.78	0.83	1.05	Hypothetical protein
QR90_RS03530	144.65	70	0.84	0.82	1.05	Hemolysin
QR90_RS09365	21	10.33	0.68	0.82	1.02	ABC ATP-binding protein
QR90_RS01280	253.16	124.44	0.74	0.8	1.02	Bifunctional pyr operon

^1^ Only CDS with probability value over 0.8 and log_2_ fold-change ≥1 are shown.

^2^ Irradiated (I) and non-irradiated (NI) expression means.

^3^ Theta: differential expression statistic calculated as (M + D)/2, where M is the log_2_-ratio of the two conditions and D is the difference in expression between conditions (including a correction for the biological variability of the corresponding feature).

^4^ Prob: probability of mixed distribution (mixed because it is calculated from features changing between conditions and invariant features).

^5^ Log_2_ fold-change.

Given that some of the genes previously reported for *Deinococcus* strains as being involved in UVR resistance [12, 13], such as DNA repair mechanisms, pigment production and efflux pumps (for *Mn*^+2^ mainly), were not present among the most differentially expressed genes, a search for orthologues of radiation-resistance genes reported from *D. radiodurans* and *D. gobiensis* was performed. All twenty-seven reported genes (e.g., citB, ddrI, phoR, phrB and mutT) were recovered with significant e-values (<0.01) but with low identity values (between 30-50% at the DNA level) and a log_2_ fold-change value not significant between the conditions tested (maximum log_2_ fold-change 0.6). In consequence, those genes were not used for further analyses.

To further characterize the 23 differentially expressed hypothetical proteins, these were analyzed for possible functional domains through HMM (Hidden Markov Models) search against the pFam database. Several domains were identified, some of which were identified as related to photosystem II (PsbP), type III secretion system lipoprotein chaperone (YscW), copper chaperone pCu(A)C, WD domain (G-beta repeat), winged helix-turn helix, and DoxX categories. Four proteins were identified as containing conserved domains of unknown function (DUF) Table 3.

**Table 3:** Predicted domains present in hypothetical proteins reported by HMMER.

GenID	Hits Found	Identifier^1^	Decription^1^	E-value^1^	Log_2_FC^2^
QR90_RS02510	1	DUF4395	Domain of unknown function	5.2E-26	0.9
QR90_RS15125	6	WD40	WD domain, G-beta repeat	7.5E-06	0.84
QR90_RS03320	1	DUF456	Domain of unknown function	1.5E-35	0.81
QR90_RS10725	1	PsbP	PsbP	7.6E-07	0.92
QR90_RS08755	1	HTH_33	Winged helix-turn helix	5E-14	0.92
QR90_RS15830	1	YscW	Type III secretion system	1.6E-07	0.92
QR90_RS03570	1	DUF4384	Domain of unknown function	1.2E-10	0.91
QR90_RS08995	1	DUF1540	Domain of Unknown Function	8.2E-08	0.82
QR90_RS02980	1	PCuAC	Copper chaperone PCu(A)C	8.4E-24	0.8
QR90_RS11035	1	DoxX	DoxX	2.9E-10	0.87

^1^ Only values for top-hit are shown.

^2^ Log_2_ fold-change.

### qRT-PCR validation

In order to validate the differential expression found with RNASeq, RT-qPCR was performed on three genes that had the highest log_2_ fold-change expression ratios: an RNA helicase (QR90_RS09640), a GntR family transcriptional regulator (QR90_RS11755) and the gene for proline dehydrogenase (QR90_RS11750). All three genes tested showed over 2-fold increase in expression (2.31, 2.14 and 2.05, respectively), thus confirming the observed RNA-seq data.

### Identification of ncRNAs

To identify additional differentially expressed features in the transcriptomes of UVC-exposed *D. swuensis* cells, and according to recently-proposed roles of ncRNAs in the rapid recovery after cellular stress, a de novo search for these regulatory entities was implemented [14]. Analysis of 3,355 intergenic regions from the D. swuensis reference genome retrieved 1,979 candidate ncRNA sequences. The criteria included a minimum cut-off for intergenic regions of 50bp and a minimum sequencing depth threshold of 6X, based on the mapping distribution, to eliminate regions with low coverage S2 Fig. These candidates were compared against covariance models (CMs) built from the Rfam database. CMs are statistical models of structurally annotated RNA multiple sequence alignments that allow a flexible search for both primary and secondary RNA structures against a known dataset [39].

The CM search reported a total of 1,598 matches, but only 290 were below a search threshold of 0.1 (parameter that describes the number of hits one can “expect” to see by chance when searching a database). These significant matches were composed by 109 RNAs involved in post-transcriptional modification (such as snRNAs/snoRNAs), and 166 regulatory RNAs (including 97 miRNAs, 20 lncRNAs, 29 cis-regulatory elements, 15 antisense and 5 CRISPR RNAs). The remaining elements included one ribozyme, three antitoxin and 11 other RNA classes. Six candidates Table 4 were significantly related to small cytoplasmic Y RNAs (Rsm Y) (e-value <0.05). The log_2_ fold-change values for the differentially expressed ncRNAs oscillated between -1.03 (for mir-234) and 1.68 (for CsrC), which doesn’t indicate a tendency towards down or up-regulation under the UV-stress condition. However, the average of probability values for all ncRNAs was only 0.26 ±0.23, whereas CsrC showed a probability of 0.79 Table 4. Although this probability is not equivalent to a p-value, the higher it is, the more likely that the difference in expression is due to the change in the experimental condition and not to chance.

**Table 4:** Significant ncRNA candidates reported by Infernal and log_2_ fold-change values for differential expression analysis.

Category	Code	E-value	Model name^4^	GC^5^	Prob	Log_2_FC^6^
Up^1^	nc0013	3.3E-05	CsrC	0.67	0.79	1.68
	nc0176	0.064	Pxr	0.5	0.45	1.46
	nc0001	0.074	ar45	0.47	0.01	1.11
	nc0011	0.01	mir-761	0.68	0.39	1.05
Reported^2^	nc0989	5.9E-05	RsmY	0.7	0.20	0.77
	nc1573	0.00092	RsmY	0.68	0.03	0.59
	nc0919	0.052	RsmX	0.58	0.08	0.40
	nc0644	0.0059	RsmY	0.75	0.33	0.38
	nc0774	0.047	RsmY	0.67	0.04	0.27
	nc0445	0.018	RsmY	0.71	0.34	0.13
Down^3^	nc0308	0.031	mir-234	0.52	0.24	-1.03

^1^ Up: Upregulated ncRNAs, log_2_ fold-change values are positive.

^2^ Reported: Significance and expression values for described RsmY family which were not differentially expressed in this study.

^3^ Down: Downregulated ncRNAs, log_2_ fold-change values are negative.

^4^ ModelName: the name of the Rfam family/model of the hit.

^5^ GC: corresponds to the fraction of G+C of the hit.

^6^ Log_2_ fold-change.

## Discussion

Natural ecosystems, and the organisms that inhabit them vary in their exposure to UVR. UVR determines the distribution and survival of microorganisms and consequently influences ecosystem dynamics and biogeochemical cycles [40]. From an evolutionary point of view, sensitivity to radiation indicates that UVR is an effective promoter of mutations, stimulating genomic variation, and could explain why high resistance is not widespread [41]. In this study, various isolates obtained from the *Espeletia* plant phyllosphere showed differences in resistance to UVR, indicating variability in their adaptability to UV exposure, despite being isolated from the same habitat. Previous reports have indicated that strains from diverse environments can differ in UVR sensitivity [41], while in other cases there can be similarity in resistance levels within a phyllosphere microbial community [42]. In our case, habitat of origin did not correlate with resistance to extreme UV-C radiation, indicating that heterogeneous microbial phenotypes coexist in these natural ecosystems.

*D. swuensis* CG1225, with >60% survival at the highest dose tested (800*J*/*m*^2^), was the most resistant isolate recovered based on our radiation resistance experiments. *Deinococcus spp*. are widely recognized as being resistant to ionizing radiation at doses that are damaging to other organisms [43]. This resistance is due to multiple mechanisms that can work synergistically to guarantee genomic integrity [38][44]. Current reported strategies include efficient DNA repair (such as RecA, Pprl, Ppr), antioxidant activities (CAT, SOD, POD, *Mn*^+ 2^), a unique cell structure (tetrad configuration for compartmentalization of DNA) [43], protection of proteins [45] and, more recently, ncRNAs [14].

In this study, we used differential gene expression analysis to identify genes involved in the cellular response of *D. swuensis* CG1225 to UVR, a strategy which has been used to study other *Deinococcus* isolates [12–14, 46]. In contrast to previous studies in which treated cells were recovered after varying lengths of time, even up to three hours post treatment [13], here the *D. swuensis* CG1225 cells were harvested right after exposure and thus provide insight regarding the immediate response to UVR exposure and irradiation stress, a snapshot of a “first quick response”. This might explain why relatively few differentially expressed genes were identified, 14 CDS with log_2_ fold-change values >1 and a probability >0.8. Although functional domains with diverse biological functions were identified in these hypothetical proteins, none of them seemed to be associated with any known UV stress-responses Table 3. It is therefore unclear what the function of several of these proteins might be and how they may contribute to the UVR stress response. The predicted genes, however, were involved in global responses to stress, such as transcription regulation and transporters involved in cellular detoxification.

The overexpressed genes support the hypothesis of an organism that turns on its transcriptional machinery, in this case as an immediate response to prepare itself for the recovery of homeostasis as response to an environmental stressor. The highest log_2_ fold-change in expression was registered for a transcription factor belonging to the GntR family, which regulates several biological processes in diverse bacterial groups, however, details regarding its specific mechanism of action remain largely uncharacterized [47]. Although this regulator protein has been associated with a decrease in resistance to stress in *D. radiodurans* [47] and *Bacillus subtilis* [48], the target genes for this transcriptional regulator remain elusive [49].

Other genes overexpressed under radiation exposure were an ABC transporter-system-related protein (ATP-binding protein), a *Mn*^+2^ transporter that has been shown to be key for ROS elimination [13, 50], and a hydrolase of the alpha/beta family. These last two genes can be potentially associated with cellular systems involved in cleaning toxic compounds produced during DNA repair. The activity of hydrolases modulating cellular redox processes has been described for many organisms [51] and in *D. radiodurans* it prevents incorporation of damaged nucleotides into DNA [49, 52]. These differentially expressed features indicate conditions that trigger the synthesis of genes and recycling of cellular components (such as chemical residues, oxidized nucleotides, etc.) from damaged biomolecules. Examples of such recycling mechanisms in *Deinococcus* come from studies showing that activity of Nudix-like hydrolases and RNA enzymes are essential for stress resistance [49, 52–54].

In this work we observed differences with respect to previous studies with *D. radiodurans* [12] and *D. gobiensis* [13]. In particular, we did not detect genes previously identified to be involved in resistance of *Deinococcus* strains, such as ddrA/ddrB genes for repair proteins, ddrC/ddrE/ddrP genes for damage response proteins and the fliY transporter. Neither these genes nor their orthologues were present among the differentially expressed genes. This discrepancy might indicate that different strategies may be involved regarding tolerance to radiation for *D. swuensis* CG1225 compared to both the widely studied *D. radiodurans* and *D. gobiensis* [55]. It could also reflect differences in experimental conditions, such as exposure to different levels of radiation [56, 57], the culture growth conditions and the amount of time allowed for cell recovery after UV exposure (from minutes to hours). In our work, the cells were exposed to a comparatively low level of radiation (considering the maximum level of resistance expressed by *D. swuensis*) and were harvested right after UVR treatment, rather than allowing longer recovery times [13]. Finally, difference in results could also be due to genome variability. The high variability in genomic organization (genome size, number of chromosomes, plasmids, etc.) in *Deinococcus* sequenced isolates has been proposed as a potential source of interesting adaptations [44]. A comparison among *D. geothermalis*, *D gobiensis* and *D. proteolyticus* showed, for example, a core genome of 1369 genes and ∼600-1700 accessory species-specific genes [44], which could harbor potential functional differences even among related species.

When analyzing the data obtained from the RNA-seq experiments, several reads could not be mapped to the reference genome used (*D. swuensis* DY59). The percentage of unmapped reads across the samples (∼10%) falls within the expected for RNA-seq experiments in which reads are mapped to a reference genome different from the evaluated isolate [13] [14], and is also consistent with the reported variability among *Deinococcus* genomes [44]. However, 30% of these unmapped reads showed identity values over 85% against the reference genome through a blast alignment, which reasserts the idea of intraspecific diversity for *Deinococcus sp*. Furthermore, an average of ∼25% of the mapped reads failed to map within annotated features. Most of these fell near (50-100pb) to the start/end positions of annotated features, suggesting that they correspond to either transcribed but un-translated regions or miss-annotated features in the genome, a reasonable explanation due to the draft version of the available reference sequence.

Given the recent reports regarding the identification of differentially expressed ncRNAs in *Deinococcus* strains, we looked for these elements in our RNA-seq data. Even though the libraries were not experimentally enriched for short ncRNAs, an exploration of reads mapping to intergenic regions allowed the recovery of some well-represented families, which can be potentially involved in the irradiation response and have not been previously reported for the reference *D. swuensis* isolate [10]. The mechanism of action for ncRNAs in radiation response is a topic of current active research, and some recent studies suggest that ncRNAs, due to their small size, might remain largely undamaged by radiation and hence be the first responders, inducing and regulating cellular function recovery [14].

Several ncRNAs were identified in this study, some potentially involved in protecting against irradiation stress. These ncRNAs included members of the RsmY and RsmX families that bind and regulate molecules, such as translational proteins RsmA/CsrA and the sigma factor RpoS (a central regulator of the general stress response) [58, 59]. Previous experiments have shown that KsgA, which belongs to the RsmA family of ncRNAs, participates in the maintenance of translational fidelity under oxidative stress in *Staphylococcus aureus* [60]. CsrC, another promising ncRNA identified here, regulates the pleiotropic gene csrA (related to RsmY and RsmX) and can cause a decrease in oxidative stress resistance in Campylobacter jejuni when damaged [61]. Other ncRNAs identified corresponded to the Mir-761 Mir-234, ar45 and Pxr families, the reported functions for these ncRNA families do not have a clear relationship with radiation resistance; understanding their roles in resistance would require additional studies.

In summary, high-throughput sequencing of RNA provided a global view of the genomic responses and shed light on potential biological strategies required for cellular adaptation [62]. Particularly, RNA-seq provides the possibility of uncovering small-scale expression changes, such as the non-common overexpressed genes and novel ncRNAs families identified for our *D. swuensis* strain isolated from the plant phyllosphere. These findings require further validation but nonetheless offer relevant insight regarding bacterial resistance to radiation stress and expand our knowledge of bacterial transcriptomic dynamics.

## Conclusion

The transcriptional behavior of *D. swuensis* under the UVR stress condition studied here revealed differentially expressed genes that differ from mechanisms commonly reported for related species and expand our understanding of UVR resistance in bacteria. The functions identified involved cell detoxification, regulation and reduction of stress by oxidation damage caused by ROS species. We also identified genes with undefined functions and previously unannotated ncRNAs families by analysis of intergenic reads under covariance models. Further studies would be needed to corroborate the observed tendency towards down-regulation of ncRNAs and the actual role played by these genes in the dynamic response after a radiation exposure event. This study contributes to the characterization of microbial biodiversity and describes potentially novel genes and small RNAs that could contribute to understanding cellular adaptations to extreme conditions and lead to potential applications, like preservation of products.

## Supporting information

S1 Fig Taxonomic assignment of the BLASTn results for unmapped reads agaist nr database. The reads were processed through MEGAN software and corresponds to controls and irradiated samples.
(TIF)

S2 Fig Histograms of read counts per sample mapping to intergenic regions. Dotted line corresponds to the selected cutoff (log10 of 0.86) implying a minimum of 6 reads per region.
(TIF)

S1 Table Primers employed for qRT-PCR. Three genes were used for evaluation, and one for normalization. T_*M*_: Melting Temperature. GC%: Percent of G+C content.
(PDF)

S2 Table Cochran’s C test for variance of groups. C:ratio of the largest variance to the sum of the variances, n:number of observations (genes) in each group, k:number of groups.
(PDF)

## References

[1] Ruiz-Pérez C a., Restrepo S, Zambrano MM. Microbial and Functional Diversity within the Phyllosphere of Espeletia sp. in an Andean High Mountain Ecosystem. Appl Environ Microbiol. 2016;82(6):AEM.02781-15. 10.1128/AEM.02781-15 26746719 PMC4784022

[2] Kwang-Woo J, Sangyong L, Yong-Sun B. Microbial radiation-resistance mechanisms. J Microbiol. 2017;55(7):499–507. 10.1007/s12275-017-7242-5 28664512

[3] Argueso JL, Westmoreland J, Mieczkowski PA, Gawel M, Petes TD, Resnick MA. Double-strand breaks associated with repetitive DNA can reshape the genome. Proc Natl Acad Sci U S A. 2008;105(33):11845–50. 10.1073/pnas.0804529105 18701715 PMC2515620

[4] Wurtmann E, Wolin SL. RNA under attack: Cellular handling of RNA damage. Crit Rev Biochem Mol Biol. 2013;31(9):34–49. 10.1080/10409230802594043 PMC2656420 19089684

[5] King B, Kesavan J, Sagripanti JL. Germicidal UV sensitivity of bacteria in aerosols and on contaminated surfaces. Aerosol Sci Technol. 2011;45(5):645–53. 10.1080/02786826.2010.550959

[6] Gabani P, Singh O V. Radiation-resistant extremophiles and their potential in biotechnology and therapeutics. Appl Microbiol Biotechnol. 2013;97(3):993–1004. 10.1007/s00253-012-4642-7 23271672

[7] Cowan DA, Ramond J, Makhalanyane TP, De Maayer P. Metagenomics of extreme environments. Curr Opin Microbiol [Internet]. 2015;25:97–102. Available from: 10.1016/j.mib.2015.05.005 26048196

[8] Gao Q, Garcia-Pichel F. Microbial ultraviolet sunscreens. Nat Rev Microbiol. 2011;9(11):791–802. 10.1038/nrmicro2649 21963801

[9] Daly MJ. A new perspective on radiation resistance based on Deinococcus radiodurans. Nat Rev Microbiol [Internet]. 2009;7(3):237–45. Available from: http://www.nature.com/doifinder/10.1038/nrmicro2073 19172147 10.1038/nrmicro2073

[10] Kim MK, Srinivasan S, Back C, Joo ES, Lee S, Jung H. Complete genome sequence of Deinococcus swuensis, a bacterium resistant to radiation toxicity. Mol Cell Toxicol. 2015;11:315–21. 10.1007/s13273-015-0031-5

[11] Lee JJ, Lee HJ, Jang GS, Yu JM, Cha JY, Kim SJ, et al Deinococcus swuensis sp. nov., a gamma-radiation-resistant bacterium isolated from soil. J Microbiol. 2013;51(3):305–11. 10.1007/s12275-013-3023-y 23812810

[12] Luan H, Meng N, Fu J, Chen X, Xu X, Feng Q, et al Genome-wide transcriptome and antioxidant analyses on gamma-irradiated phases of Deinococcus radiodurans R1. PLoS One. 2014;9(1). 10.1371/journal.pone.0085649 PMC3900439 24465634

[13] Yuan M, Chen M, Zhang W, Lu W, Wang J, Yang M, et al Genome sequence and transcriptome analysis of the radioresistant bacterium Deinococcus gobiensis: Insights into the extreme environmental adaptations. PLoS One. 2012;7(3):1–11. 10.1371/journal.pone.0034458 PMC3314630 22470573

[14] Tsai C, Liao R, Chou B, Contreras LM. Transcriptional Analysis of Deinococcus radiodurans Reveals Novel Small RNAs That Are Differentially Expressed under Ionizing Radiation. Appl Environ Microbiol [Internet]. 2015;81(5):1754–64. Available from: http://aem.asm.org/lookup/doi/10.1128/AEM.03709-14 25548054 10.1128/AEM.03709-14 PMC4325154

[15] Sonnleitner E, Romeo A, Blaesi U Small regulatory RNAs in Pseudomonas aeruginosa. 10.4161/rna.19231 22336763

[16] Wassarman KM. Small RNAs in bacteria: Diverse regulators of gene expression in response to environmental changes. Cell. 2002;109(2):141–4. 10.1016/s0092-8674(02)00717-1 12007399

[17] Kowalski MP, Krude T. Functional roles of non-coding Y RNAs. Int J Biochem Cell Biol [Internet]. 2015;66:20–9. Available from: 10.1016/j.biocel.2015.07.003 26159929 PMC4726728

[18] Chen X, Sim S, Wurtmann EJ, Feke A, Wolin SL. Bacterial noncoding Y RNAs are widespread and mimic tRNAs. Rna [Internet]. 2014;20(11):1715–24. Available from: http://rnajournal.cshlp.org/lookup/doi/10.1261/rna.047241.114 25232022 10.1261/rna.047241.114 PMC4201824

[19] Chen X, Wurtmann EJ, Van Batavia J, Zybailov B, Washburn MP, Wolin SL. An ortholog of the Ro autoantigen functions in 23S rRNA maturation in D. radiodurans. Genes Dev. 2007;21(11):1328–39. 10.1101/gad.1548207 17510283 PMC1877746

[20] Bodenhausen N, Horton MW, Bergelson J. Bacterial Communities Associated with the Leaves and the Roots of Arabidopsis thaliana. PLoS One. 2013;8(2). 10.1371/journal.pone.0056329 23457551 PMC3574144

[21] Paulino-Lima IG, Azua-Bustos A, Vicuña R, González-Silva C, Salas L, Teixeira L, et al Isolation of UVC-Tolerant Bacteria from the Hyperarid Atacama Desert, Chile. Microb Ecol. 2013;65(2):325–35. 10.1007/s00248-012-0121-z 23001596

[22] Bolger AM, Lohse M, Usadel B. Trimmomatic: A flexible trimmer for Illumina sequence data. Bioinformatics. 2014;30(15):2114–20. 10.1093/bioinformatics/btu170 24695404 PMC4103590

[23] Kopylova E, Noé L, Touzet H. SortMeRNA: Fast and accurate filtering of ribosomal RNAs in metatranscriptomic data. Bioinformatics. 2012;28(24):3211–7. 10.1093/bioinformatics/bts611 23071270

[24] Pruesse E, Quast C, Knittel K, Fuchs BM, Ludwig W, Peplies J, et al SILVA: A comprehensive online resource for quality checked and aligned ribosomal RNA sequence data compatible with ARB. Nucleic Acids Res. 2007;35(21):7188–96. 10.1093/nar/gkm864 17947321 PMC2175337

[25] Liao Y, Smyth GK, Shi W. The Subread aligner: fast, accurate and scalable read mapping by seed-and-vote. Nucleic Acids Res. 2013;41(10):108–24. 10.1093/nar/gkt214 PMC3664803 23558742

[26] Liao Y, Smyth GK, Shi W. FeatureCounts: An efficient general purpose program for assigning sequence reads to genomic features. Bioinformatics. 2014;30(7):923–30. 10.1093/bioinformatics/btt656 24227677

[27] Huson DH, Beier S, Flade I, Górska A, El-Hadidi M, Mitra S, et al MEGAN Community Edition—Interactive Exploration and Analysis of Large-Scale Microbiome Sequencing Data. PLoS Comput Biol. 2016;12(6):1–12. 10.1371/journal.pcbi.1004957 PMC4915700 27327495

[28] Tarazona S, Furió-Tarí P, Turrá D, Di Pietro A, Nueda MJ, Ferrer A, et al Data quality aware analysis of differential expression in RNA-seq with NOISeq R/Bioc package. Nucleic Acids Res. 2015;43(21):1–15. 26184878 10.1093/nar/gkv711 PMC4666377

[29] Sha Y, Phan J, May W. Effect of low-expression gene filtering on detection of differentially expressed genes in RNA-seq data. Eng Med Biol Soc (EMBC), 2015 37th Annu Int Conf IEEE (pp 6461-6464). 2015;70(12):773–9. 10.1109/EMBC.2015.7319872 PMC4983442 26737772

[30] Mistry J, Finn RD, Eddy SR, Bateman A, Punta M. Challenges in homology search: HMMER3 and convergent evolution of coiled-coil regions. Nucleic Acids Res. 2013;41(12). 10.1093/nar/gkt263 23598997 PMC3695513

[31] Finn RD, Coggill P, Eberhardt RY, Eddy SR, Mistry J, Mitchell AL, et al The Pfam protein families database: Towards a more sustainable future. Nucleic Acids Res. 2016;44(D1):D279–85. 10.1093/nar/gkv1344 26673716 PMC4702930

[32] Kalvari I, Argasinska J, Quinones-Olvera N, Nawrocki EP, Rivas E, Eddy SR, et al Rfam 13.0: shifting to a genome-centric resource for non-coding RNA families. Nucleic Acids Res [Internet]. 2017;(November):1–8. Available from: http://academic.oup.com/nar/article/doi/10.1093/nar/gkx1038/4588106 29112718 10.1093/nar/gkx1038 PMC5753348

[33] Pruitt KD, Tatusova T, Maglott DR. NCBI reference sequences (RefSeq): A curated non-redundant sequence database of genomes, transcripts and proteins. Nucleic Acids Res. 2007;35(SUPPL. 1):501–4. 10.1093/nar/gkl842 PMC1716718 17130148

[34] Nawrocki EP, Eddy SR. Infernal 1.1: 100-fold faster RNA homology searches. Bioinformatics. 2013;29(22):2933–5. 10.1093/bioinformatics/btt509 24008419 PMC3810854

[35] Pfaffl MW. A new mathematical model for relative quantification in real-time RT-PCR. Nucleic Acids Res [Internet]. 2001;29(9):e45 Available from: http://www.ncbi.nlm.nih.gov/pubmed/11328886 11328886 10.1093/nar/29.9.e45 PMC55695

[36] Chen F, Sorek R, Hugenholtz P, Lindquist EA, Froula JL, He S, et al Validation of two ribosomal RNA removal methods for microbial metatranscriptomics. Nat Methods. 2010;7(10):807–12. 10.1038/nmeth.1507 20852648

[37] Conesa A, Madrigal P, Tarazona S, Gomez-Cabrero D, Cervera A, McPherson A, et al A survey of best practices for RNA-seq data analysis. Genome Biol. 2016;17(1):1–19. 10.1186/s13059-016-1047-4 26813401 PMC4728800

[38] Slade D, Radman M. Oxidative Stress Resistance in Deinococcus radiodurans. Microbiology and Molecular Biology Reviews [Internet]. 2011;75(1):133–191 p. Available from: http://mmbr.asm.org/cgi/doi/10.1128/MMBR.00015-10 21372322 10.1128/MMBR.00015-10 PMC3063356

[39] Barquist L, Burge SW, Gardner PP. Studying RNA homology and conservation with infernal: From single sequences to RNA families. Curr Protoc Bioinforma. 2016;2016:12.13.1–12.13.25. 10.1002/cpbi.4 PMC5010141 27322404

[40] Arrieta JM, Weinbauer MG, Gerhard J, Mari S. Interspecific Variability in Sensitivity to UV Radiation and Subsequent Recovery in Selected Isolates of Marine Bacteria. Appl Environ Microbiol. 2000;66(4):1468–73. 10.1128/aem.66.4.1468-1473.2000 10742228 PMC92009

[41] Gascón J, Oubiña A, Pérez-Lezaun A, Urmeneta J. Sensitivity of selected bacterial species to UV radiation. Curr Microbiol. 1995;30(3):177–82. 10.1007/bf00296205 7765851

[42] Sundin GW, Jacobs JL. Ultraviolet radiation (UVR) sensitivity analysis and UVR survival strategies of a bacterial community from the phyllosphere of field-grown peanut (Arachis hypogeae L.). Microb Ecol. 1999;38(1):27–38. 10.1007/s002489900152 10384007

[43] Wei LI, Yun MA, Fangzhu X, Shuya HE. Ionizing Radiation Resistance in Deinococcus Radiodurans. Adv Nat Sci. 2014;7(2):6–14.

[44] Gerber E, Bernard R, Castang S, Chabot N, Coze F, Dreux-Zigha A, et al Deinococcus as new chassis for industrial biotechnology: Biology, physiology and tools. J Appl Microbiol. 2015;119(1):1–10. 10.1111/jam.12808 25809882 PMC4682472

[45] Daly MJ. Death by protein damage in irradiated cells. DNA Repair (Amst) [Internet]. 2011;11(1):12–21. Available from: 10.1016/j.dnarep.2011.10.024 22112864

[46] Ozsolak F, Platt AR, Jones DR, Reifenberger JG, Sass LE, McInerney P, et al Direct RNA sequencing. Nature [Internet]. 2009;461(7265):814–8. Available from: http://www.ncbi.nlm.nih.gov/pubmed/19776739 19776739 10.1038/nature08390

[47] Dulermo R, Onodera T, Coste G, Passot F, Dutertre M, Porteron M, et al Identification of new genes contributing to the extreme radioresistance of Deinococcus radiodurans using a Tn5-based transposon mutant library. PLoS One. 2015;10(4):1–26. 10.1371/journal.pone.0124358 PMC4401554 25884619

[48] Lord DM, Uzgoren Baran A, Soo VWC, Wood TK, Peti W, Page R. McbR/YncC: Implications for the mechanism of ligand and DNA binding by a bacterial gntr transcriptional regulator involved in biofilm formation. Biochemistry. 2014;53(46):7223–31. 10.1021/bi500871a 25376905 PMC4245980

[49] Agapov AA, Kulbachinskiy A. V. Mechanisms of stress resistance and gene regulation in the radioresistant bacterium Deinococcus radiodurans. Biochem [Internet]. 2015;80(10):1201–16. Available from: http://link.springer.com/10.1134/S0006297915100016 26567564 10.1134/S0006297915100016

[50] Liesa M, Qiu W, Shirihai OS. Mitochondrial ABC transporters function: The role of ABCB10 (ABC-me) as a novel player in cellular handling of reactive oxygen species. Biochim Biophys Acta—Mol Cell Res [Internet]. 2012;1823(10):1945–57. Available from: 10.1016/j.bbamcr.2012.07.013 PMC3808996 22884976

[51] Tong L, Lee S, Denu JM. Hydrolase regulates NAD+ metabolites and modulates cellular redox. J Biol Chem. 2009;284(17):11256–66. 10.1074/jbc.M809790200 19251690 PMC2670130

[52] Makarova KS, Aravind L, Daly MJ, Koonin E V. Specific expansion of protein families in the radioresistant bacterium Deinococcus radiodurans. Genetica. 2000;108(1):25–34. 10.1023/a:1004035424657 11145417

[53] Makarova KS, Omelchenko M V., Gaidamakova EK, Matrosova VY, Vasilenko A, Zhai M, et al Deinococcus geothermalis: The pool of extreme radiation resistance genes shrinks. PLoS One. 2007;2(9). 10.1371/journal.pone.0000955 17895995 PMC1978522

[54] Ott E, Kawaguchi Y, Kölbl D, Chaturvedi P, Nakagawa K, Yamagishi A, et al Proteometabolomic response of Deinococcus radiodurans exposed to UVC and vacuum conditions: Initial studies prior to the Tanpopo space mission. PLoS One. 2017;12(12):1–25. 10.1371/journal.pone.0189381 PMC5731708 29244852

[55] Ohtani N, Tomita M, Itaya M. An extreme thermophile, Thermus thermophilus, is a polyploid bacterium. J Bacteriol. 2010;192(20):5499–505. 10.1128/JB.00662-10 20729360 PMC2950507

[56] Dib J, Motok J, Zenoff VF, Ordoñez O, Farías ME. Occurrence of resistance to antibiotics, UV-B, and arsenic in bacteria isolated from extreme environments in high-altitude (above 4400 m) Andean wetlands. Curr Microbiol. 2008;56(5):510–7. 10.1007/s00284-008-9103-2 18330637

[57] Guerrero-Beltrán JA, Barbosa-Cánovas G V. Advantages and Limitations on Processing Foods by UV Light. Food Sci Technol Int. 2004;10(3):137–47. 10.1177/1082013204044359

[58] Hengge-Aronis R. Signal transduction and regulatory mechanisms involved in control of the sigma(S) (RpoS) subunit of RNA polymerase. Microbiol Mol Biol Rev [Internet]. 2002;66(3):373–95, table of contents. Available from: http://www.pubmedcentral.nih.gov/articlerender.fcgi?artid=120795&tool=pmcentrez&rendertype=59 12208995 10.1128/MMBR.66.3.373-395.2002 PMC120795

[59] Rasis M, Segal G. The LetA-RsmYZ-CsrA regulatory cascade, together with RpoS and PmrA, post-transcriptionally regulates stationary phase activation of Legionella pneumophila Icm/Dot effectors. Mol Microbiol. 2009;72(4):995–1010. 10.1111/j.1365-2958.2009.06705.x 19400807

[60] Kyuma T, Kizaki H, Ryuno H, Sekimizu K, Kaito C. 16S rRNA methyltransferase KsgA contributes to oxidative stress resistance and virulence in Staphylococcus aureus. Biochimie [Internet]. 2015;119:166–74. Available from: 10.1016/j.biochi.2015.10.027 26545800

[61] Fields JA, Thompson SA. Campylobacter jejuni CsrA mediates oxidative stress responses, biofilm formation, and host cell invasion. J Bacteriol. 2008;190(9):3411–6. 10.1128/JB.01928-07 18310331 PMC2347403

[62] Wang Z, Gerstein M, Snyder M. RNA-Seq: a revolutionary tool for transcriptomics. Nat Rev Genet. 2009;10(1):57–63. 10.1038/nrg2484 19015660 PMC2949280

