# Supplementary figures and images for: Computational search for UV radiation resistance strategies in Deinococcus swuensis isolated from Paramo ecosystems
Source: PLoS One. 2019 Dec 2;14(12):e0221540. doi: 10.1371/journal.pone.0221540 (PMC6886795; doi:10.1371/journal.pone.0221540)

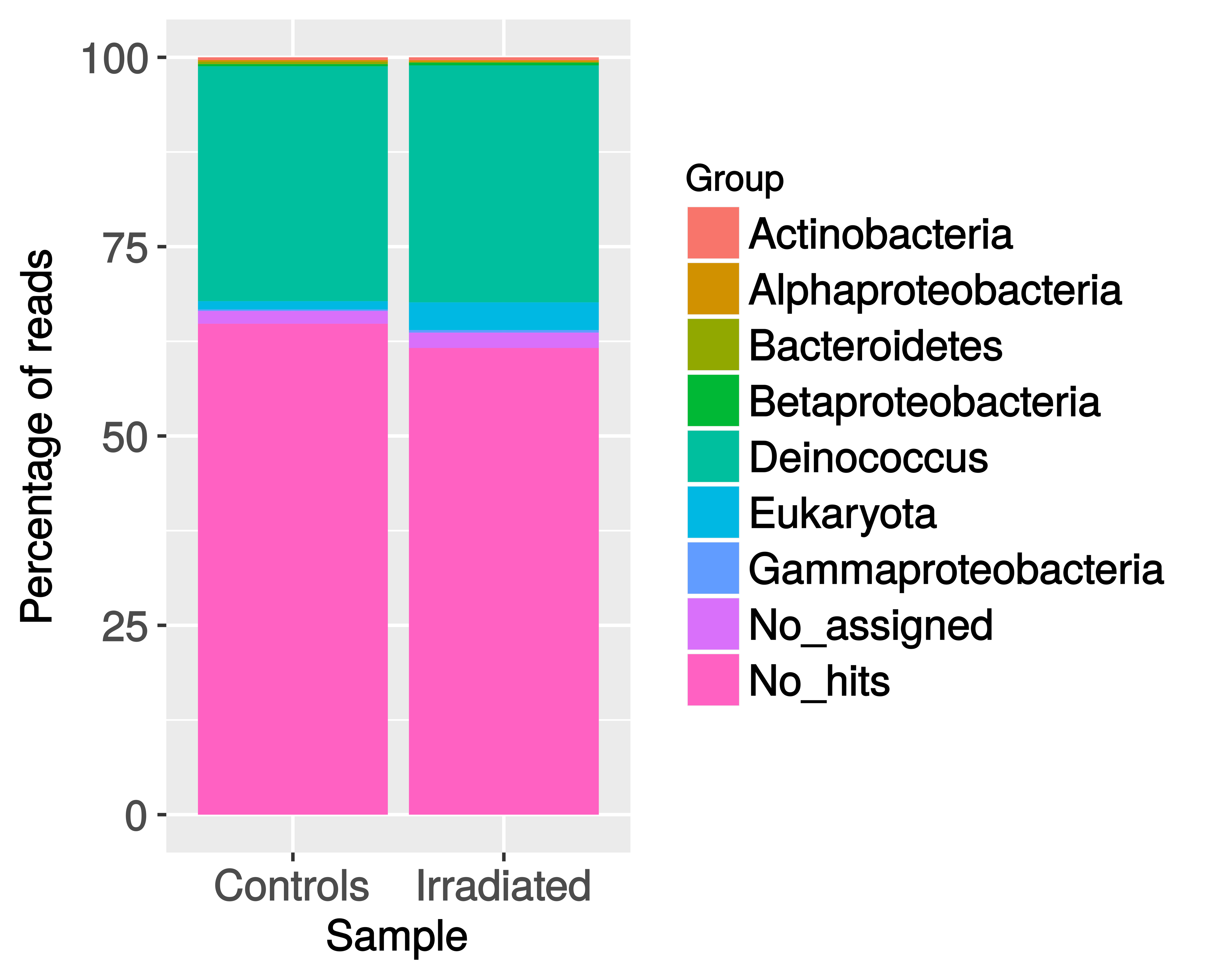

Supplement: S1 Fig — The reads were processed through MEGAN software and corresponds to controls and irradiated samples. (TIF) [file pone.0221540.s001.tif]

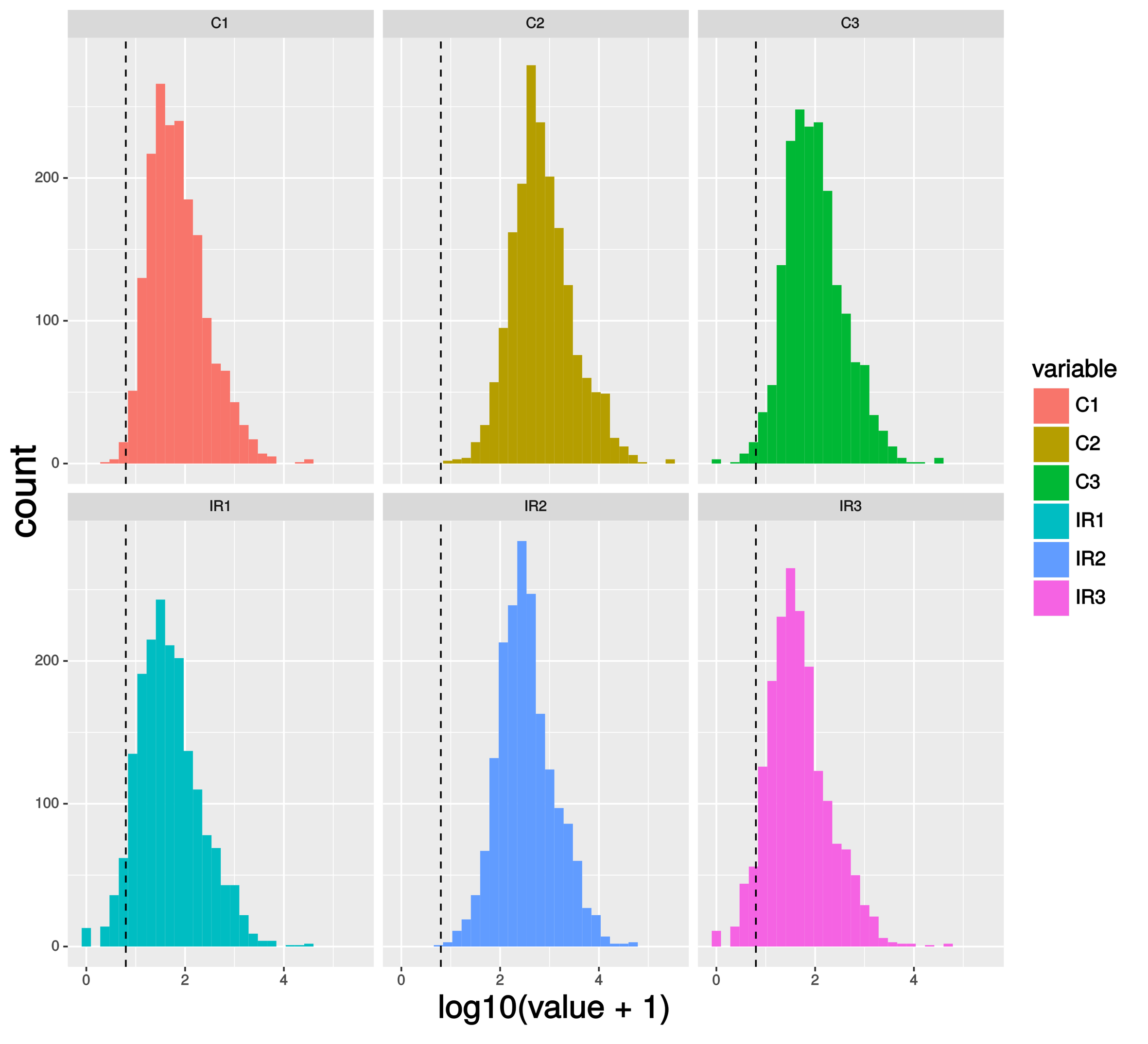

Supplement: S2 Fig — Dotted line corresponds to the selected cutoff (log10 of 0.86) implying a minimum of 6 reads per region. (TIF) [file pone.0221540.s002.tif]
