# Supplementary material for: Computational search for UV radiation resistance strategies in Deinococcus swuensis isolated from Paramo ecosystems
Source: PLoS One. 2019 Dec 2;14(12):e0221540. doi: 10.1371/journal.pone.0221540 (PMC6886795; doi:10.1371/journal.pone.0221540)
Supplement: S1 Table — Three genes were used for evaluation, and one for normalization. TM: Melting Temperature. GC%: Percent of G+C content. (PDF) [file pone.0221540.s003.pdf]

Table 1: **Primers employed for qRT-PCR.** Three genes were used for evaluation, and one for normalization. T_M: Melting Temperature. GC%: Percent of G+C content

Type	GenID	Amplicon length (pb) [1]	Direction	T_M	GC%	Sequence (5'->3')
Evaluated	QR90_RS11755[1]	202	Forward	62.0	52.6	GCTGGACGGTGAGATTGTT
			Reverse	62.0	50.0	TTCCTCACGCACAATGTAGG
	QR90_RS09640[2]	209	Forward	62.1	50	TGATTCACGGCGAGAGATTG
			Reverse	62.2	55	CAGTTCGGGCAGTTCCTTAG
	QR90_RS11750[3]	223	Forward	62	52.4	GATCAGACCTTGGAGCAGTTC
			Reverse	62	55	CGTAGACCAGTTTGCGGTAG
Normalizer	QR90_RS09970[4]	210	Forward	62	40.9	AACGTTTATTGACGCGAAACAG
			Reverse	62	50	AGGATCAGCCAGTCGTAGAA

[1] GntR family transcriptional regulator.
[2] RNA helicase.
[3] Proline dehydrogenase.
[4] Succinate dehydrogenase.
