# Supplementary material for: Computational search for UV radiation resistance strategies in Deinococcus swuensis isolated from Paramo ecosystems
Source: PLoS One. 2019 Dec 2;14(12):e0221540. doi: 10.1371/journal.pone.0221540 (PMC6886795; doi:10.1371/journal.pone.0221540)
Supplement: S2 Table — C:ratio of the largest variance to the sum of the variances, n:number of observations (genes) in each group, k:number of groups. (PDF) [file pone.0221540.s004.pdf]

Table 1: Cochrans C test for variance of groups: C:ratio of the largest variance to the sum of the variances, n:number of observations (genes) in each group, k:number of groups.

	Controls			Irradiated		
**Sample**	**C1**	**C2**	**C3**	**IR1**	**IR2**	**IR3**
**Variance**	0.6701	0.9226	0.6896	0.6284	0.6849	0.6022

C = 0.21122, n = 3223, k = 6, p-value <2.2e-16
